# Stem Cell and Exosome Treatment for Organ Health and Longevity

**DOI:** 10.3390/ijms27209174

**Published:** 2026-10-16

**Authors:** Chadwick Prodromos, Ruby Del Villar, Andrew Striegel, Gerard Pena, Rohan Dixit, Amy Xu

**Affiliations:** The Prodromos Stem Cell Institute, 1714 Milwaukee Ave., Glenview, IL 60025, USA

**Keywords:** stem cells, exosomes, anti-aging, organ rejuvenation, longevity, sarcopenia

## Abstract

Aging is associated with inflammation and is the greatest risk factor for chronic disease. Mesenchymal stem cells (MSCs) and exosomes have anti-inflammatory, pro-healing, and immunomodulatory effects that make them appealing candidates to reverse tissue and organ aging, improve overall organ function, and promote longevity. We hypothesized that treatment with MSCs and/or exosomes would improve the function of healthy mammalian organ systems and tissues, extend lifespan, and that treatment with stem cells from younger animals would have a greater rejuvenative effect than those from older animals. Through a review of the PubMed-indexed scientific literature, we sought to determine whether there is sound preclinical and/or clinical evidence to support these hypotheses. 34 preclinical studies were chosen as they demonstrated that stem cells and/or exosomes can restore bone density, improve muscle strength, significantly rejuvenate multiple organ systems, and increase longevity. Stem cell treatment appears to increase longevity, and both stem cell and exosome treatments are effective in rejuvenating and improving tissue and organ function in mammalian systems. Stem cells can extend overall mammalian lifespan. Transplantation of bone-marrow derived mesenchymal stem cells can restore bone density in mice. MSCs have a regenerative effect on aging muscles in mice. Both stem cells and exosomes have a rejuvenative effect on animal organ systems. Stem cells and exosomes improve organ function in the absence of pathological states.

## 1. Introduction

Aging is the gradual deterioration of the physiological functions necessary for survival and fertility [1]. Genomic instability, telomere attrition, epigenetic alterations, loss of proteostasis, chronic inflammation, deregulated nutrient sensing, mitochondrial dysfunction, cellular senescence, stem cell exhaustion, and altered intercellular communication are universal hallmarks of aging across different organisms [2]. According to the Centers for Disease Control and Prevention (CDC), chronic diseases are the leading cause of disability, death, and illness in America [3]. Aging is the greatest risk factor for chronic diseases, including heart disease, Alzheimer’s Disease, cancer, and chronic kidney disease [4]. Therefore, anti-aging, rejuvenative therapies would be greatly beneficial in decreasing the chronic disease population burden.

Mesenchymal stem cells (MSCs) are multipotent cells that can differentiate into a variety of different types of human cells, including, but not limited to, bone, cartilage, fat, muscle, and nerve [5,6]. MSCs can be isolated from various tissue sources, such as umbilical cord, placenta, dental pulp, bone marrow, and fat [7,8].

MSCs and exosomes have anti-inflammatory, pro-healing, and immunomodulatory effects [9,10]. MSCs exert these effects by secreting growth factors and cytokines such as transforming growth factor-1 (TGF-β1), prostaglandin E2 (PGE2), and hepatocyte growth factor (HGF) which assist with modulating the body’s immune response [11]. MSCs also secrete extracellular vesicles (EVs) such as exosomes, microvesicles, and apoptotic bodies [11]. These EVs, exosomes in particular are small vesicles, approximately 100–200 nanometers in diameter and have emerged as potential therapeutic options in various disorders, as they contain various proteins, miRNA, and DNA [11]. Stem cells have been shown to be effective in treating a variety of disorders. They are also often promulgated as effective in improving overall health and increasing lifespan in otherwise clinically healthy individuals. However, there are limited evidence-based studies examining the effects of stem cells and exosomes on longevity and the potential for improved health in nonpathological human organs. While there is a general agreement that stem cells and exosomes have potential to be helpful to organ aging and longevity, there has not yet been a comprehensive, evidence-based evaluation of this thought. Through a review of the PubMed-indexed scientific literature, we sought to determine whether there is scientific evidence to support the use of MSCs and exosomes to reverse tissue and organ aging, improve function, and increase longevity and overall health in the absence of specific disease states. We hypothesized that preclinical and clinical data would show that treatment with MSCs and/or exosomes would result in measurable improvement in the functioning of healthy mammalian organ systems and tissues. We further hypothesized that treatment with stem cells from younger animals would have a greater rejuvenative effect than treatment with stem cells from more mature animals. We conclude that MSCs and exosomes rejuvenate mammalian organ systems, restoring them to a less aged state, and that stem cells improve longevity specifically.

An indexed literature search was conducted on PubMed to examine the anti-aging and rejuvenative effects of MSCs, exosomes, or EVs on healthy and diseased mammalian organs, including the brain, kidney, liver, thymus, spleen, skin/hair, lungs, gut, and heart. Besides aiming for studies that were published post-2000, there were no specific criteria when deciding which studies were to be included, nor a specific number of studies that were aimed for. From this search, we chose a total of 34 different preclinical studies using stem cells and/or exosomes that were performed in mice, rats, or macaques. We believe that these 34 studies were an adequate amount for this review to showcase the anti-aging effects of MSCs, exosomes, and EVs. One of the 34 studies was published in *Circulation* and was available only as an abstract. Figure 1 displays a basic flowchart of the PubMed search that was conducted containing all organs researched in addition to studies we used that observed longevity and sarcopenia applications. Table 1 contains all 34 studies discussed in this manuscript in addition to information about if MSCs or exosomes were used, the source of the MSCs/exosomes, the animal model used, and what condition/organs that were studied.

**Table 1:** Table summarizing all 34 individual studies discussed.

Reference	MSC/Exosome/EV/Media/Stem Cells	MSC/Exosome Source	Model	Organ/Condition Observed
Shen et al. 2011 [12]	MSC	Bone marrow	Mice	Longevity/Aging
Yamaza et al. 2009 [13]	MSC	Bone marrow	Mice	Longevity/Aging
Dorronsoro et al. 2021 [14]	MSCs/Media/EVs	Bone marrow	Mice	Longevity/Aging
Wang et al. 2023 [15]	MSCs	Umbilical Cord	Mice	Sarcopenia
Piao et al. 2022 [16]	MSCs	Umbilical Cord	Mice	Sarcopenia
Zhang et al. 2023 [17]	Exosomes	Unspecified	Mice	Brain
Liang et al. 2025 [18]	MSCs	Umbilical Cord	Mice	Brain
Tfilin et al. 2023 [19]	MSCs	Bone marrow	Mice	Brain
Cao et al. 2017 [20]	MSCs	Umbilical Cord	Mice	Brain
El-Akabawy et al. 2023 [21]	MSCs	Bone marrow	Rats	Brain
Yang et al. 2011 [22]	MSCs	Bone marrow	Mice	Kidneys
Xing et al. 2025 [23]	Exosomes	Umbilical Cord	Mice	Kidneys
Yu et al. 2025 [24]	Exosomes	Umbilical Cord	Mice	Kidneys
Liu et al. 2022 [25]	MSCs	Adipose	Mice	Longevity/Aging
Xing et al. 2023 [26]	MSCs	Deciduous teeth	Mice	Liver
Ling et al. 2023 [27]	Exosomes	Umbilical Cord	Mice	Liver
Wang et al. 2012 [28]	MSCs	Bone marrow	Rats	Liver
Wang et al. 2020 [29]	MSCs	Bone marrow	Rats	Thymus/spleen
Wang et al. 2018 [30]	EVs	Serum	Mice	Thymus/spleen
Liu et al. 2026 [31]	MSCs	Bone marrow	Mice/Monkeys	Spleen
Xu et al. 2020 [32]	EVs	Adipose	Mice	Skin
Shi et al. 2021 [33]	EVs	Gum tissue	Mice	Skin/Heart
Li et al. 2022 [34]	Exosomes	Adipose	Mice	Hair
Yang et al. 2021 [35]	MSC	Bone marrow	Macaques	Lungs
Bi et al. 2025 [36]	Exosomes	Embryonic Stem Cell	Mice	Kidneys/Liver/Lungs/Spleen/Skin
Wang et al. 2024 [37]	MSCs	Umbilical Cord	Rats	Lungs/Kidneys/Gut/Brain/Spleen/Liver/Heart
Su et al. 2023 [38]	EVs	Bone marrow	Mice	Lungs
Wang et al. 2025 [39]	MSCs	Placenta	Mice	Gut
Ocansey et al. 2022 [40]	Exosomes	Umbilical Cord	Mice	Gut
Gu et al. 2021 [41]	Exosomes	Bone marrow	Mice	Gut
Long et al. 2025 [42]	EVs	Adipose	Mice	Gut
Zhang et al. 2015 [43]	MSCs	Bone marrow	Rats	Heart
Olivares et al. 2004 [44]	MSCs	Bone marrow	Rats	Heart
El-Akabawy et al. 2024 [45]	Stem cells	Dental pulp tissue	Rats	Heart

**Figure 1:**
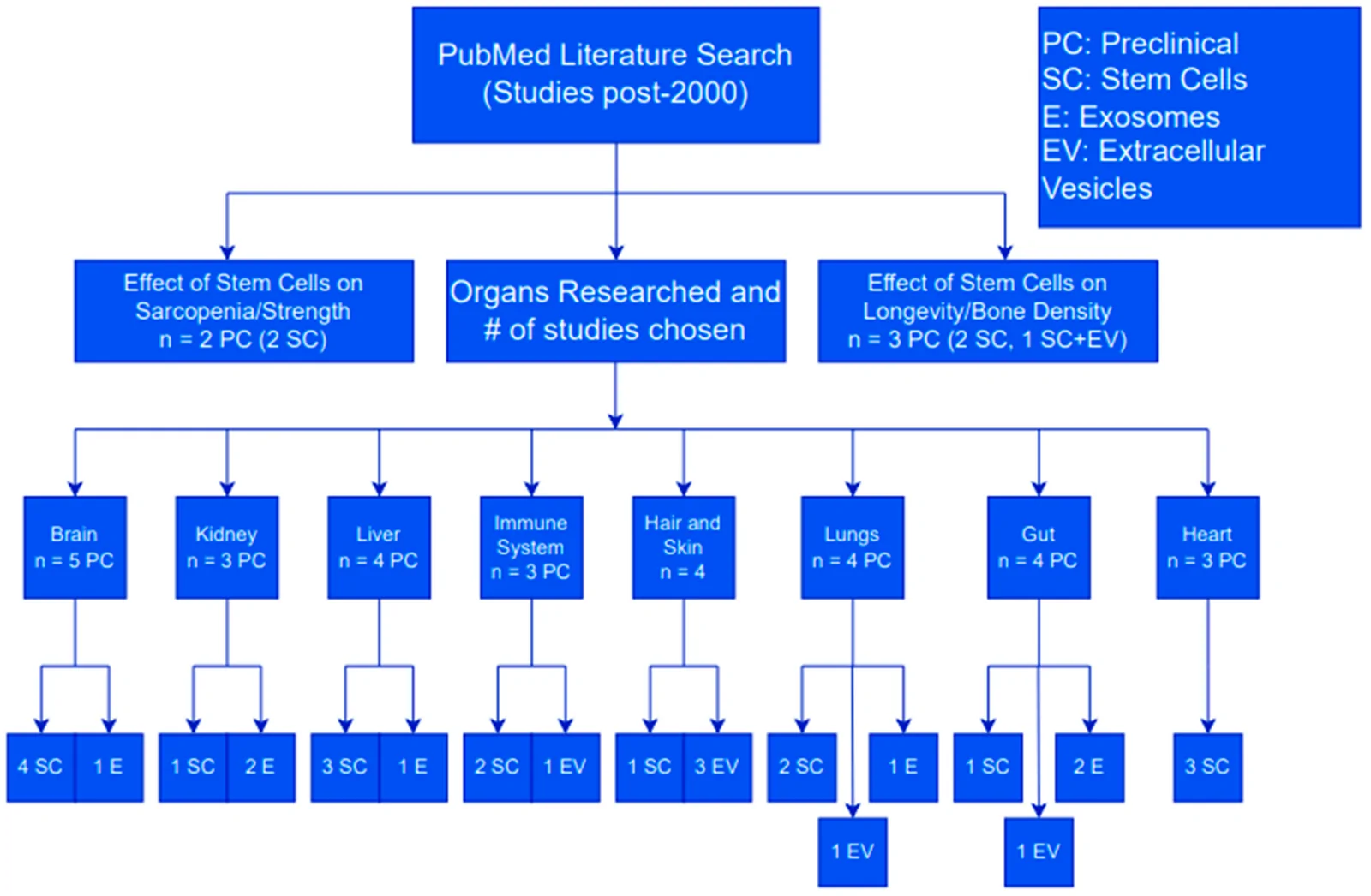
Overview of PubMed literature search examining the anti-aging and rejuvenative capabilities of stem cells, exosomes, and extracellular vesicles on 8 different organ systems, as well as the effects of stem cells on sarcopenia, longevity, and bone density. Only studies published post-2000 were looked at. No other specific criteria was used when choosing studies. There were 34 individual studies that were chosen to be included in this manuscript; however, the figure presents 35 studies due to one study being used for both the liver and hair/skin (ref. [25]). Diagram was created using https://app.diagrams.net/ (accessed on 6 October 2026).

## 2. Results

### 2.1. Longevity/Bone Density

In a preclinical study, bone marrow-derived MSCs (BMSCs) from young donors were transplanted into old mice, resulting in restoration of bone density. Unexpectedly, the old mice transplanted with young BMSCs also had a longer lifespan compared to the control mice and mice that received BMSCs from old donors. The mean lifespan of control mice was 765 days, while the mean lifespan of mice that received young BMSC transplants was 890 days. The mean lifespan of mice transplanted with old BMSCs was 789 days [12]. These results suggest that transplantation of young BMSCs can extend lifespan. The young stem cell-transplanted mice exhibited a 16% increase in lifespan. For an adult American, this would translate to 12 years of additional life from the young cells, but only 2 years from the old cells. Another study assessed the use of BMSCs in 6-month-old immunocompromised mice. The results showed that the BMSC-transplanted mice had an average extension of lifespan that was 33.4% greater than that of control mice. To assess this survival, the authors used the Mantel–Haenszel test. In addition, aging phenotypes present in control mice were absent in all surviving BMSC-transplanted mice [13]. The exact mechanism by which MSCs exert their benefits for longevity is still unclear, but there are some studies that propose potential mechanisms of action. One preclinical study involved the administration of BMSCs, BMSC-conditioned media, and BMSC extracellular vesicles (EVs) in young mice. This study proposed a paracrine effect involving EVs that are secreted by BMSCs [14]. These EVs contain miRNA that can enter surrounding cells and reduce several markers associated with cell senescence such as PTEN, MYC, and IGF-1R [14]. The study also discusses how some senescent cells develop a senescence-associated secretory phenotype (SASP) which involves more pro-inflammatory cytokines being secreted, and the authors observed decreases in IL-6 and IL-1β after treatment, which in turn decreases the SASP [14]. This decrease in inflammatory cytokines and SASP indicate that the MSCs could be preventing these senescent factors from affecting surrounding cells to potentially halt the progression of cellular aging [14].

### 2.2. Sarcopenia/Strength

Sarcopenia is the progressive loss of muscle mass and strength associated with aging [46]. In one study [15], clinical-grade human umbilical cord-derived MSCs (hUC-MSCs) were administered to two mouse models of age-associated sarcopenia (AAS): SAMP8 (a senescence-accelerated model) and D-galactose-induced mice. It was found that administration of hUC-MSCs improved muscle strength and restored muscle morphology in AAS mouse models. Additionally, the SAMP8 control mice that received phosphate-buffered saline instead of hUC-MSCs showed significantly reduced gastrocnemius muscle cross-sectional area and diameter. However, the cross-sectional area and diameter were restored in the group that received hUC-MSCs. These results suggest that MSCs may have a regenerative effect on aging muscles in mice and would likely increase strength in humans. This is consistent with our clinical experience, in which patients treated with stem cells often report increased strength after treatment. The mechanisms by which MSCs can contribute to improving muscle volume and strength were proposed in another study involving the administration of hUC-MSCs in a senescence-accelerated sarcopenia murine model [16]. The hUC-MSCs are thought to secrete bioactive molecules such as hepatocyte growth factor (HGF) and vascular endothelial growth factor (VEGF) which activate adenosine monophosphate-activated protein kinase (AMPK) signaling [16]. This also leads to aiding mitochondrial dysfunction and the activation of peroxisome proliferator-activated receptor-γ coactivator1-α (PGC1-α), which is a protein that is heavily involved in mitochondrial biogenesis in skeletal muscle [16]. In addition, proteins involved in cell apoptosis such as Caspase-3,8 are suppressed by the effects of the hUC-MSCs which can help with the prevention of further muscle loss [16]. All these effects are thought to work simultaneously to ameliorate skeletal muscle atrophy and dysfunction.

### 2.3. Brain

There is no more important health and longevity rejuvenation target than the brain. Stem cells do not effectively cross a healthy blood–brain barrier (BBB) when injected intravenously. However, exosomes have been shown in at least 41 animal studies to enter the brain cerebrospinal fluid through the cribriform plate at the top of the nose. They enter neurons, bypass the blood–brain barrier, and have been shown to be effective in our clinical studies treating ALS [47], MS, Parkinson’s disease, and stroke. We have also seen significant benefits in a majority of patients without brain disease but with early cognitive decline, manifesting as short-term memory loss and decreased focus due to aging.

The mechanism of action is not completely clear, but one study showed that exosomes secreted by MSCs (MSC-exos) decreased mitochondrial apoptosis and reduced oxidative stress in the brains of aged mice [17]. Thus, intranasal exosome treatment can improve age-related decreases in neuronal function. We also have generated EEG data showing improved brain status after brain-related treatment [48].

In one pre-clinical study, age-related cognitive decline was ameliorated after stem cell treatment. The findings showed that “hUC-MSCs attenuate microglial senescence, reduce LD accumulation, and inhibit the NF-κB-SREBP1 signaling pathway, ultimately mitigating neuroinflammation and neuronal damage” [18]. Another pre-clinical study found that IV administration of polarized MSCs reduced serum levels of pro-aging chemokines, leading to positive effects on hippocampal neurogenesis and behavior [19]. This supports the idea that MSCs can positively affect the brain directly. In a separate study in mice that used the conventional aging model induced by D-galactose, it was found that 3 months of treatment with hUC-MSCs improved learning, memory, and synaptic plasticity in the hippocampus [20]. An additional study by El-Akabawey et al. examined the effects of BMSCs in D-galactose-induced rat brains. The results showed that BMSCs “prevented many of the locomotion and coordination deficits present in the D-galactose-induced aging rat model by restoring dopaminergic (DA) system function, protecting atrophic DA neurons in the substantia nigra (SN), inducing antioxidative effects, and secreting neurotrophic growth factors” [21].

### 2.4. Kidney

Glomerular filtration rate (GFR) decreases normally after age 40, usually without clinical symptoms. However, over time, symptoms can appear, and the kidneys become more vulnerable to loss of function from disorders such as diabetes and high blood pressure as their function declines. Animal studies have shown that there are regenerative effects of bone-marrow derived cells on the kidney [22]. In our clinical studies, we have also seen improved kidney function in patients with stage 1 and stage 2 kidney failure, which mimics age-related kidney decline. Exosome treatment has also shown efficacy in aging mouse models. According to one preclinical study in mice [23], human umbilical cord mesenchymal stem cell-derived exosomes (hUC-MSC-exos) reduced inflammatory infiltration, tubular degeneration, and interstitial fibrosis in kidney tissue. Additionally, aging markers were reduced (SA-β-gal, p16INK4a, and γH2AX) in the hUC-MSC-exos treatment group. In a different study, Yu et al. conducted phosphoproteomic analysis on aging kidneys in mice with and without hUC-MSC-exo treatment [24]. The authors found through their analysis that lamin A/C phosphorylation was reduced in aged kidneys, and after hUC-MSC-exo treatment, lamin A/C phosphorylation was increased [24]. Lamin A/C supports nucleus structure which can be affected with age, and this phosphorylation support can suppress p53-p21 senescence signaling and SASP leading to the attenuation of kidney aging, which is exactly what the authors found [24].

### 2.5. Liver

The liver, among all organ systems, shows the greatest capacity to rejuvenate—a fact known even to the ancient Greeks in the legend of Prometheus [49]. Stem cells have shown the ability to rejuvenate and restore diseased livers. There is also animal data showing that stem cell treatment can alleviate inflammatory infiltration and fiber deposition present in the livers of aged mice [25]. Another study in naturally aging mice using stem cells from human exfoliated deciduous teeth (SHED) showed “After 6 months of continuous SHED injections, the liver proteomic pattern was reversed from senescent (~30 months) to a youthful state (~3 months), accompanied with an upregulation of hepatocyte marker genes, anti-aging protein Klotho, and a global improvement of liver functional pathway proteins” [26]. Another preclinical study in naturally aging mouse livers [27] showed that hUC-MSC-exos can improve structural disorder and decrease markers of senescence in aging livers. Additionally, hUC-MSC-exos were shown to decrease glycerophospholipids, palmitoyl-glycerols, and eicosanoid derivatives associated with toxicity and inflammation. As mentioned earlier, MSCs can secrete HGF, which can also be contained in secreted exosomes. One study investigated this deeper and found that HGF may be beneficial for aging livers, as the authors found that HGF secreted from BMSCs suppress the TLR4/MyD88/NF-κB signaling pathway leading to the inhibition of hepatic stellate cells, which are key contributors to liver fibrosis [28]. Since liver fibrosis is more prone to occur with age, this suggests that MSCs and exosomes can indirectly halt the progression of liver fibrosis and inflammation [28].

### 2.6. Immune System—Thymus and Spleen

The thymus and spleen are the organs principally responsible for the immune system. Animal studies have shown that BMSCs significantly improve tissue damage in the aging spleen and thymus; one study by Wang et al. Showed that BMSCs may improve aging organs by influencing cytokines, oxidative stress, and P21/PCNA [29]. One preclinical mouse study investigated the effects of extracellular vesicles (EVs) isolated from young donor mouse serum on the thymuses of naturally aged mice (18–20 months) [30]. It was found that “the attenuation of inflammaging after treatment with EVs from young serum partially contributed to the rejuvenation of thymic aging, which is characterized by partially reversed thymic involution, enhancement of negative selection signals, and reduced auto reactions in the periphery.” After injection, the older mice had increased thymus size, increased thymocyte numbers, and increased expression of ΔNp63 and FoxN1, which are transcription factors that regulate thymic epithelial stem-cell proliferation [30]. The authors propose that the upregulation of these transcription factors contributes to improved thymic microstructure leading to the thymus exhibiting a younger thymic phenotype [30]. In a separate spleen study conducted by Liu et al., injections of healthy human MSCs were given to older mice and monkeys and their spleens were compared to untreated control mice and monkeys [31]. After treatment, it was found that the spleens of the older mice increased in size, had more splenic cells, and a restoration of the splenic stromal cell network [31]. For the monkeys, ultrasound imaging revealed increased spleen length compared to control monkeys [31]. The authors hypothesize that the main driver behind this rejuvenation is VEGFA, a growth factor in the VEGF family that is secreted by MSCs [31]. The proposed mechanism is that secreted VEGFA binds to VEGFR3 marginal reticular cells (MRCs), which are stromal cells found in the spleen [31]. This binding activates the Erk and Akt signaling pathways which induces more MRC proliferation and regeneration leading to rejuvenation [31].

### 2.7. Hair and Skin

Hair thins with age to varying degrees in all genders. There is data showing that BMSC treatment greatly improves the swollen, inactivated, and reduced number of hair follicles present in older mice, as well as the diminished number of subcutaneous fibers [25]. For skin, one preclinical study involved exposing mice to UVB light multiple times a week for 8 weeks to induce skin aging [32]. These mice were also given adipose MSC-EVs subcutaneously during light exposure sessions [32]. Compared to untreated mice, treated mice saw better skin improvements such as fewer wrinkles, less skin roughness, increased epithelial cell proliferation, fewer senescent cells, and improved epidermal thickness [32]. As to why MSCs and exosomes may be beneficial for skin rejuvenation, one preclinical study looked at performing intravenous administration of EVs derived from gingiva-derived MSCs in aged mice [33]. In this study, the authors discovered that the EVs may aid aged skin by suppressing mTOR/S6K signaling, which plays a critical role in oxidative stress-induced cell senescence and aging [33]. As for hair, Li et al. proposed that adipose MSC-EVs may contribute to hair regrowth by downregulating miR-22 and the TNF-α signaling pathway since they play roles in hair follicle loss [34]. The authors also mention that the EVs may also activate the Wnt/β-catenin signaling pathway which can aid in accelerating hair cycle progression [34].

### 2.8. Lungs

Stem cell infusion has been shown to be effective in our patients with COPD and asthma. In addition, there is strong animal data showing lung rejuvenation and anti-aging effects with IV infusion. One study concluded: “BMMSC treatment inhibited age-associated lung tissue degeneration” [35]. In another study [36], human embryonic stem cell-derived exosomes (hESC-exos) containing miR-302b reversed the accumulation of senescent cells in the kidneys, liver, lungs, spleen, and skin of aging mice. Additionally, chronic inflammation in the circulation of the aged mice was reduced. Wang et al. conducted a study with aged rats, and they were given repeated intravenous administrations of UC-MSCs to see the effects on multiple organs [37]. One of the observed organs were the lungs, and the authors found that naturally aged lungs exhibit enlarged alveolar ducts, more fibrous tissue, fewer epithelial cells, and increased inflammation, and UC-MSC treatments helped to ameliorate all these issues [37]. Why MSCs and MSC exosomes are beneficial for aging lungs can be explained by a study conducted by Su et al. [38]. Despite not being a study that observed naturally aged lungs and looked at a murine acute lung injury model, the authors propose that MSCs may help lungs by delivering miR-181a-5p, which is naturally found in secreted EVs from MSCs [38]. This was found by giving mice intravenous injections of either normal MSC-EVs, MSC-EVs with upregulated miR-181a-5p, and MSC-EVs with silenced miR-181a-5p, and the authors found that both the normal MSC-EVs and upregulated MSC-EVs resulted in reduced lung inflammation and reduced alveolar protein leakage, while silenced MSC-EVs did not exhibit these benefits [38]. This led the authors to believe that miR-181a-5p could be one of the driving factors to healing/rejuvenating lungs by downregulating PTEN which then activates pSTAT5 leading to reduced lung inflammation and injury [38].

### 2.9. Gut

Stem cells have been shown to reduce intestinal inflammation and correct “leaky” gut syndrome. This has been demonstrated in a preclinical study from China by Wang et al. [39]. Since normal intestines also become more inflamed and function less effectively with age, this rejuvenative effect would be expected to be beneficial even for people without a specific disease diagnosis. Many such individuals simply experience indigestion and other GI-related symptoms. MSC-exos have also demonstrated anti-inflammatory effects in the gut, helping to mitigate colitis in animal models [40]. A separate preclinical study in dextran sodium sulfate (DSS)-induced colitis mice found that MSC-exos might protect intestinal barrier function and relieve inflammation through the transport of miR-181a in vivo [41]. The reason why exosomes/EVs may be beneficial for aging guts was proposed by Long et al., where they administered adipose-derived EVs from young mice donors to both young and old mice [42]. The guts of aged mice compared to young mice had more intestinal inflammation, dysfunctional gut microbiome, and increased gut permeability [42]. After treatment, the older mice exhibited lower gut permeability and a gut microbiome composition that reflected more that of a younger mouse microbiome [42]. The authors propose that adipose EVs from younger mice contain retinoic acid, which is delivered to intestinal B cells leading to increased IgA production. This results in improved microbiome homeostasis and improved gut permeability [42].

### 2.10. Heart

One preclinical study by Zhang et al. found that “BMMSCs show antisenescence action on aging neonatal rat ventricular cells and heart and improve cardiac function in aging rats” [43]. A separate preclinical study in healed infarcted rat hearts examined whether BMSCs transplanted into the myocardium could improve cardiac function [44]. Electrocardiography and echocardiography showed that BMSC-treated animals had significant improvements in cardiac function compared with rats treated with cell culture medium. An additional study by El-Akabawey et al. showed the anti-aging effects of dental pulp stem cells on healthy rat hearts [45]. The transplanted stem cells were able to differentiate into cardiomyocytes and “improve cardiac function, upregulate Sirt1 expression, exert antioxidative effects, modulate connexin-43 expression, attenuate cardiac histopathological alterations, and have anti-senescent and anti-apoptotic effects.” The anti-aging effects of human exosomes on 24-month-old rat cardiomyocytes have also been studied. The results suggest that cardiosphere-derived cell exosomes from young human donors (CDC-exos) “increase telomerase activity, lengthen telomeres, and decrease cell senescence in old rat heart cells.” As for potential molecular mechanisms, the same study from Zhang et al. proposed that MSCs may aid the aging heart by decreasing reactive oxygen species leading to the decrease in p53 and p21 and less cardiomyocyte senescence, which is similar to the kidney study Yu et al. conducted [24,43]. This was found by injecting young donor BMSCs in the hearts of older rats which led to reduced oxidative stress, decreased SA-β-gal activity in the hearts (an indication of reduced senescence), reduced hypertrophy and fibrosis, and improved heart function [43].

## 3. Discussion

This review demonstrates, for the first time to our knowledge, that stem cells and, separately, exosomes can partially reverse aging-related changes in healthy tissues and organs, including the brain, kidney, liver, thymus, spleen, skin, lungs, hair, gut, and heart. Additionally, MSCs have been shown to restore bone density, improve muscle strength, and promote regeneration of aging muscles in mice by restoring the cross-sectional area and diameter of gastrocnemius muscle fibers. Clinical results from our institute indicate that stem cell injections into the scalp, as well as intravenous administration, have resulted in increased hair thickness for well over a year in most patients.

This is the first article, to our knowledge, to separately assess the rejuvenative effects of MSCs and exosomes on otherwise healthy mammalian tissue. Other studies have shown efficacy in improving diseased tissue but have not specifically demonstrated that MSCs and exosomes can be beneficial in the absence of pathological states. One current research gap is the limited amount of clinical evidence evaluating the rejuvenative potential of MSCs and exosomes across different organ systems, as well as their effects on longevity. Despite there being studies that have presented evidence for how stem cells and exosomes enact their effects, their mechanisms of action are also both still not confirmed. Though, it is widely thought from the studies that were analyzed that stem cells have a paracrine effect by releasing growth factors, cytokines, and extracellular vesicles including exosomes which enter target cells.

While current literature favors the positive effects of MSCs and MSC-derived exosomes, there are also downsides depending on MSC quality and control. When MSCs are expanded in vitro and/or are transplanted in vivo, they divide and with each division, telomeres can shorten in addition to DNA damage occurring leaving the MSCs in a more senescent state [8]. In this state, the proliferative and therapeutic effects of the MSCs are diminished, and they are driven to a more pro-inflammatory SASP [8,50]. This causes MSCs to potentially become detrimental and cause more tissue damage due to an increase in the secretion of pro-inflammatory factors and cytokines [8], which can also negatively affect EV/exosome cargo [51]. This was demonstrated in a 2024 study conducted by Boulestrau et al. where EVs derived from senescent human adipose MSCs were transplanted in a murine osteoarthritis model [52]. These senescent EVs did not prevent cartilage damage, joint calcification, and bone degradation, and even induced more inflammation in the joints [52]. To prevent these complications, especially when conducting MSC or MSC-derived exosome transplantation in humans, cell manufacturers should aim to keep MSC passage numbers low, with a 2026 review from Fang et al. recommending P3-P5 [53].

Even when using MSCs or MSC-derived exosomes that are within the recommended passage numbers, there is still some risk involved, despite most studies showing positive results. One study in 2024 conducted by Yang et al. involved intravenous infusions of hUC-MSC-derived EVs in healthy mice to assess their coagulative properties [54]. The authors found that high doses of hUC-MSC-derived EVs did lead to the formation of multiple thrombi in lung tissue causing increased mortality rates [54]. A different 2024 study by Kwon et al. also resulted in thromboembolism and death in the majority of a cohort of young, healthy mice after intravenous infusions of canine-derived adipose-MSCs [10]. These studies show that both MSCs and MSC-derived EVs do pose some risk of causing adverse events such as thromboembolism. To prevent such events from occurring, treatment providers need to have the correct dose and infusion rate.

Future developments in this field are likely to focus on more in-human clinical trials. While there have been clinical trials that have observed the use of MSCs in frail humans with some positive results [55,56,57,58], the evidence is still quantitatively lackluster, and more trials still need to be conducted. Aging is arguably the most important and resource-intensive public health problem globally. Since there is no other proven anti-aging treatment, the demonstration that MSCs and exosomes are effective in slowing and partially reversing aging and improving lifespan is of great potential public health significance. This study is therefore of great clinical significance in demonstrating for the first time evidence that stem cell treatment, by increasing the function of healthy organ systems, is an effective anti-aging treatment.

## 4. Conclusions

Both stem cells and exosomes have been shown to significantly rejuvenate healthy organ systems in preclinical studies, restoring their function to a more youthful state. Longevity was also increased in two studies. Beyond their use in treating specific disease states, stem cell and exosome therapies appear justified as interventions to improve human tissue and organ function, as well as promote longevity.

## Data Availability

No new data were created or analyzed in this study. Data sharing is not applicable to this article.
